# The accuracy of HPV genotyping in isolation and in combination with CD4 and HIV viral load for the identification of HIV‐infected women at risk for developing cervical cancer

**DOI:** 10.1002/cam4.3785

**Published:** 2021-02-19

**Authors:** Chandrika J. Piyathilake, Suguna Badiga, Greer A. Burkholder, Shuko Harada, James L. Raper

**Affiliations:** ^1^ Department of Nutrition Sciences The University of Alabama at Birmingham (UAB) Birmingham AL USA; ^2^ Department of Medicine/Division of Infectious Diseases The University of Alabama at Birmingham (UAB) Birmingham AL USA; ^3^ Department of Pathology The University of Alabama at Birmingham (UAB) Birmingham AL USA

**Keywords:** CD4, cervical cancer, cervical cytology, HPV, NPV, PPV, VL

## Abstract

**Background:**

Human papillomavirus (HPV) genotype testing has limited utility to identify human immunodeficiency virus‐infected (HIV+) women's risk for developing cervical cancer (CC) due to high positivity rate of high‐risk (HR) HPVs. We investigated the accuracy of HPV testing in isolation/in combination with CD4 and HIV viral load (VL) to identify HIV+ women at risk for developing CC.

**Methods:**

Study consisted of 344 HIV+ women on combination antiretroviral therapy (cART), tested for cervical cytology/HPV using the Cobas test and had data on absolute CD4 count and VL measurements. We calculated the positive predictive value (PPV) and negative predictive value (NPV) of HPV testing, pre‐, post‐cART, and current CD4 and VL in isolation and in combinations to identify those with or free of higher than atypical squamous cells of unknown significance (ASCUS+) or low‐grade intraepithelial lesions (LSIL+).

**Results:**

HPV test in combination with pre‐/post‐cART or current CD4 counts and VL had higher PPVs compared to HPV test alone for identifying ASCUS+ or LSIL+. PPV of HPV‐CD4 combinations yielded higher PPVs compared to HPV‐VL combinations. The NPVs with pre‐, post‐cART, or current CD4 count and VL in isolation or in combinations were comparable to that of HPV test alone.

**Conclusions:**

Our results provide a more accurate tool for managing HIV+ women by combining Cobas HPV with CD4 and VL, especially those who had an undesirable pre‐cART CD4 and VL status. Our results also indicate the usefulness of CD4 and VL measurements to identify those at lower risk in the absence of HPV testing.

## Graphical abstract

The purpose of the study was to investigate the accuracy of HPV genotype testing in isolation and in combination with CD4 and HIV viral load (VL) for the identification of women infected with HIV at risk for developing cervical cancer. Our results indicated the importance of knowing CD4 and VL status in addition to HPV infection status in identifying women infected with HIV who are at risk for developing CC.

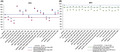

## 1 INTRODUCTION

Individuals infected with human immunodeficiency virus (HIV+) and treated with combination antiretroviral therapy (cART) have reduced risk of acquired immune deficiency syndrome (AIDS) and dramatically prolonged the survival of people with HIV in developed countries.^1^ Women with HIV (WWH) in the United States^2^ and worldwide^3^, ^4^, ^5^ are significantly more likely to acquire infection with human papillomaviruses (HPVs), the causative agent for developing cervical intraepithelial neoplasia (CIN) and cervical cancer (CC), an AIDS‐defining cancer.^6^, ^7^ Since women represent almost 50% of all adults living with HIV worldwide,^8^ it is imperative that WWH do not succumb to CC, largely a preventable disease.

The U.S. Food and Drug Administration approved the Cobas HPV test for use in co‐testing with cervical Papanicolaou smear or as a standalone test for screening women >25 years of age. However, these historical cervical screening approaches and biomarkers of HPV‐related carcinogenesis may have limited utility for that purpose in WWH because of the high positivity rate of high‐risk (HR) HPVs.^9^ The strong independent relationship between infection with HR‐HPVs and low CD4 count and higher risk for prevalent/incident abnormal cervical cytology in WWH is well established.^2^, ^10^, ^11^, ^12^ HIV‐mediated immune dysfunction could lead to persistent and more intense cervical HPV infections and their lower clearance rate,^13^ facilitating the development of precursor lesions (abnormal pap and/or CIN) for CC.^14^ HIV infection may also directly promote HPV‐associated carcinogenesis at the molecular level since the HIV‐encoded Tat protein can enhance the expression of viral oncogenes.^15^

Based on these factors, it is logical to assume that HPV testing in combination with CD4 cell count and HIV viral load (VL) which are surrogate markers for immune system function and the degree of CD4 cell destruction are likely to more accurately identify women at risk for CC. To our knowledge, however, this is the first study that investigated the accuracy of HPV genotype testing in isolation and in combination with CD4 and VL for the identification of WWH at risk for developing CC.

## 2 STUDY POPULATION

Study population consisted of 344 Caucasian American (CA) or African American (AA) WWH who have been followed for an average of 15 years and recently seen at the University of Alabama at Birmingham (UAB) 1917 HIV Outpatient Clinic from 2018 to 2020 where they were screened for abnormal cervical cytology and reflex tested for HPV status using the Cobas HPV assay (Roche Molecular Diagnostics). All women have been on cART for ≥6 months and had ≥three CD4 count and VL measurements after the cART initiation date. We used CD4 counts and VL measures at all visits since the time of HIV diagnosis and at the date closest to each patient's cervical cytology diagnosis and Cobas HPV testing. At the clinic, a nurse practitioner collected exo‐ and endocervical samples using clinic standard collection protocols. PreservCyt vials containing cells labeled at the bedside were transported to the UAB cytopathology laboratory for cytological testing and the molecular diagnostic laboratory for HPV testing.

## 3 VARIABLES AND OUTCOMES

The main variables of interest for the current study were race, age at cervical cytology diagnosis/Cobas HPV testing referred to as current age, HPV results, and pre‐cART, post‐cART, and current absolute CD4 count and VL measures. Pre‐cART CD4 count was the calculated mean CD4 count of all visits prior to cART initiation and was categorized based on the cut points <100, <200, or <350 cell/mm^3^. Post‐cART CD4 count was summarized as proportion of time CD4 count was <100 or <200 or <350 cells/mm^3^, categorized as higher (≥50% or ≥30%) or lower frequency (<50% or <30%) based on the 50% or 30% cut point. Current CD4 count was the measurement closest to the date of the cytology diagnosis/HPV test results, categorized by the cut points <100 or <200 or <350 cells/mm^3^. Pre‐cART VL was the mean HIV RNA VL per ml measures prior to the initiation of cART categorized based on the threshold cutoffs ≥10,000, ≥50,000, and ≥100,000 copies/mL. The current and post‐cART VL was categorized as detectable at the level of ≥20 copies/mL, considering 20 copies/mL is the lowest threshold for detecting plasma HIV RNA for the test used at UAB for this population. Post‐cART VL was summarized as the proportion (50% or 30% cut point) of the time VL is detectable.

Of the 344 WWH, 98 were diagnosed with atypical squamous cells of unknown significance (ASCUS) or higher‐grade (ASCUS+ includes ASCUS n = 48, low‐grade intraepithelial lesions [LSIL] n = 41, high‐grade squamous intraepithelial lesions [HSIL] n = 9), and 246 were negative for intraepithelial lesions or malignancy (NILM, referred to as free of ASCUS+ or LSIL+). Women were categorized for HPV status based on Cobas HPV test results. Cobas® HPV test (Roche Molecular Diagnostics) uses an automated sample preparation by Cobas 4800 system and concomitant amplification and identification of 13 HR‐HPV genotypes and identifies the presence of HPV16 and HPV18 individually using Cobas Z 480 analyzer. Based on Cobas results, we observed the following categories; positive for HPV 16 and negative for HPV 18 and other HR‐HPVs (n = 4), positive for HPV 18 and negative for HPV 16 and other HR‐HPVs (n = 2), negative for HPV 16 but positive for HPV 18 and other HR‐HPVs (n = 6), negative for HPV 18 but positive for HPV 16 and other HR‐HPVs (n = 14), negative for HPV 16 and HPV 18 but positive for other HR‐HPVs (n = 85), positive for HPV 16, HPV 18 and other HR‐HPVs (n = 2) and negative for HPV 16 HPV 18 and all other HR‐HPVs (n = 231). Because of the small numbers in some categories, we combined women positive for either HPV 16 or 18 or other HR‐HPVs as positive for any HR‐HPV (n = 113) and all those negative for HPV 16, 18, and other HR‐HPVs as negative for any HR‐HPV (n = 231). Studies with larger sample size are required to test associations of interest with specific HPV genotypes.

## 4 DATA ANALYSIS

The characteristics of the population variables were summarized as percentages. We assessed the positive predictive value (PPV) and negative predictive value (NPV) value of Cobas HPV test results (positive or negative), alone and in specific combinations with pre‐cART, post‐cART, and current CD4 count and VL measurements for identifying those with or free of ASCUS+ or LSIL+ in the entire population and stratified by race.

## 5 RESULTS

The median age of the study population was 48 years. The majority (83%) of WWH were AAs and 17% were CAs. About 28% of women were diagnosed with ASCUS+ while 17% were diagnosed with LSIL+. About 33% of women tested positive for any HR‐HPV detected by the Cobas HPV test. The number and percentages of women in various categories of individual test results and their specific combinations are shown in Table 1.

**TABLE 1:** The number and percentage of the study population by the Cobas HPV test results, CD4 counts, and HIV VL and their combinations (N = 344)

Test	N (%)
HR‐HPV+	98 (33%)
Pre‐cART CD4	
Pre‐cART CD4 <100 cells/mm^3^	57 (17%)
Pre‐cART CD4 <200 cells/mm^3^	92 (27%)
Pre‐cART CD4 <350 cells/mm^3^	147 (43%)
Post‐cART CD4	
≥50% of the time post‐cART CD4 <100 cells/mm^3^	20 (6%)
≥50% of the time post‐cART CD4 <200 cells/mm^3^	41 (12%)
≥50% of the time post‐cART CD4 <350 cells/mm^3^	103 (30%)
≥30% of the time post‐cART CD4 <100 cells/mm^3^	32 (9%)
≥30% of the time post‐cART CD4 <200 cells/mm^3^	74 (22%)
≥30% of the time post‐cART CD4 <350 cells/mm^3^	139 (40%)
Current CD4	
Current CD4 <100 cells/mm^3^	19 (6%)
Current CD4 <200 cells/mm^3^	35 (10%)
Current CD4 <350 cells/mm^3^	72 (21%)
Pre‐cART VL	
Pre‐cART VL ≥10,000 copies/mL	149 (43%)
Pre‐cART VL ≥50,000 copies/mL	87 (25%)
Pre‐cART VL ≥100,000 copies/mL	59 (17%)
Post‐cART VL	
≥50% of the time post‐cART VL detectable	182 (53%)
≥30% of the time post‐cART VL detectable	235 (68%)
Current VL	
Current VL detectable	124 (36%)
HR‐HPV & pre‐cART CD4	
HR‐HPV+ & pre‐cART CD4 <100 cells/mm^3^	21 (6%)
HR‐HPV+ & pre‐cART CD4 <200 cells/mm^3^	34 (10%)
HR‐HPV+ & pre‐cART CD4 <350 cells/mm^3^	51 (15%)
HR‐HPV & post‐cART CD4	
HR‐HPV+ & ≥50% of the time post‐cART CD4 <100 cells/mm^3^	15 (4%)
HR‐HPV+ & ≥50% of the time post‐cART CD4 <200 cells/mm^3^	22 (6%)
HR‐HPV+ & ≥50% of the time post‐cART CD4 <350 cells/mm^3^	51 (15%)
HR‐HPV+ & ≥30% of the time post‐cART CD4 <100 cells/mm^3^	17 (5%)
HR‐HPV+ & ≥30% of the time post‐cART CD4 <200 cells/mm^3^	36 (10%)
HR‐HPV+ & ≥30% of the time post‐cART CD4 <350 cells/mm^3^	64 (19%)
HR‐HPV & current CD4	
HR‐HPV+ & current CD4 <100 cells/mm^3^	11 (3%)
HR‐HPV+ & current CD4 <200 cells/mm^3^	20 (6%)
HR‐HPV+ & current CD4 <350 cells/mm^3^	38 (11%)
HR‐HPV & pre‐cART VL	
	
HR‐HPV+ & pre‐cART VL ≥ 10,000 copies/mL	51 (15%)
HR‐HPV+ & pre‐cART VL ≥50,000 copies/mL	32 (9%)
	
HR‐HPV+ & pre‐cART VL ≥ 100,000 copies/mL	22 (6%)
HR‐HPV & post‐cART VL	
HR‐HPV+ & ≥50% of the time post‐cART VL detectable	73 (21%)
HR‐HPV+ & ≥30% of the time post‐cART VL detectable	87 (25%)
HR‐HPV & current VL	
HR‐HPV+ & current VL detectable	59 (17%)
HR‐HPV & pre‐cART VL & pre‐cART CD4	
HR‐HPV+ & pre‐cART VL ≥10,000 copies/mL & pre‐cART CD4 <100 cells/mm^3^	19 (6%)
HR‐HPV+ & pre‐cART VL ≥50,000 copies/mL & pre‐cART CD4 <100 cells/mm^3^	18 (5%)
HR‐HPV+ & pre‐cART VL ≥100,000 copies/mL & pre‐cART CD4 <100 cells/mm^3^	16 (5%)
HR‐HPV+ & pre‐cART VL ≥10,000 copies/mL & pre‐cART CD4 <200 cells/mm^3^	25 (7%)
HR‐HPV+ & pre‐cART VL ≥50,000 copies/mL & pre‐cART CD4 <200 cells/mm^3^	23 (7%)
HR‐HPV+ & pre‐cART VL ≥100,000 copies/mL & pre‐cART CD4 <200 cells/mm^3^	20 (6%)
HR‐HPV+ & pre‐cART VL ≥10,000 copies/mL & pre‐cART CD4 <350 cells/mm^3^	35 (10%)
HR‐HPV+ & pre‐cART VL ≥50,000 copies/mL & pre‐cART CD4 <350 cells/mm^3^	28 (8%)
HR‐HPV+ & pre‐cART VL ≥100,000 copies/mL & pre‐cART CD4 <350 cells/mm^3^	20 (6%)
HR‐HPV & post‐cART VL & post‐cART CD4	
HR‐HPV+ & ≥50% of the time post‐cART VL detectable & ≥50% of the time post‐cART CD4 <100 cells/mm^3^	14 (4%)
HR‐HPV+ & ≥50% of the time post‐cART VL detectable & ≥50% of the time post‐cART CD4 <200 cells/mm^3^	21 (6%)
HR‐HPV+ & ≥50% of the time post‐cART VL detectable & ≥50% of the time post‐cART CD4 <350 cells/mm^3^	40 (12%)
HR‐HPV+ & ≥30% of the time post‐cART VL detectable & ≥50% of the time post‐cART CD4 <100 cells/mm^3^	14 (4%)
HR‐HPV+ & ≥30% of the time post‐cART VL detectable & ≥50% of the time post‐cART CD4 <200 cells/mm^3^	21 (6%)
HR‐HPV+ & ≥30% of the time post‐cART VL detectable & ≥50% of the time post‐cART CD4 <350 cells/mm^3^	45 (13%)
HR‐HPV+ & ≥50% of the time post‐cART VL detectable & ≥30% of the time post‐cART CD4 <100 cells/mm^3^	16 (5%)
HR‐HPV+ & ≥50% of the time post‐cART VL detectable & ≥30% of the time post‐cART CD4 <200 cells/mm^3^	29 (8%)
HR‐HPV+ & ≥50% of the time post‐cART VL detectable & ≥30% of the time post‐cART CD4 <350 cells/mm^3^	51(15%)
HR‐HPV+ & ≥30% of the time post‐cART VL detectable & ≥30% of the time post‐cART CD4 <100 cells/mm^3^	16 (5%)
HR‐HPV+ & ≥30% of the time post‐cART VL detectable & ≥30% of the time post‐cART CD4 <200 cells/mm^3^	32 (9%)
HR‐HPV+ & ≥30% of the time post‐cART VL detectable & ≥30% of the time post‐cART CD4 <350 cells/mm^3^	57 (17%)
HR‐HPV & current VL & current CD4	
HR‐HPV+ & current VL detectable & current CD4 <100 cells/mm^3^	10 (3%)
HR‐HPV+ & current VL detectable & current CD4 <200 cells/mm^3^	18 (5%)
HR‐HPV+ & current VL detectable & current CD4 <350 cells/mm^3^	30 (9%)

**FIGURE 1:**
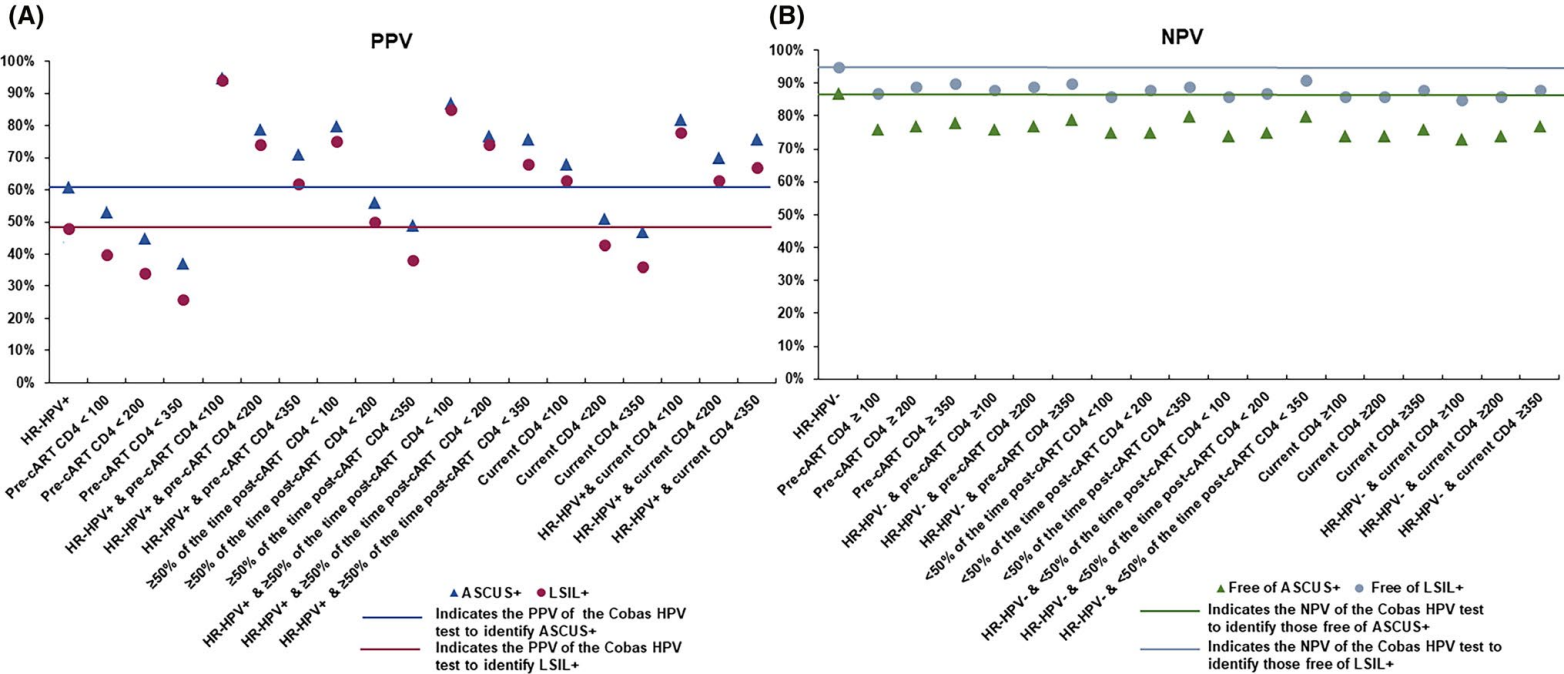
PPV (A) and NPV (B) of the Cobas HPV test alone and in combination with specific categories of pre‐cART, post‐cART, and current CD4 for identifying those diagnosed with or free of ASCUS+ or LSIL+

The PPV for identifying ASCUS+ or LSIL+ when Cobas HPV test was positive was 61% and 48%, respectively. The NPV for identifying 72% of women diagnosed with NILM against ASCUS+ or LSIL+ was 87% and 95%, respectively. As shown in Figure 1A and Tables S1 and S2, all of the pre‐cART, post‐cART, and current CD4 cut points in isolation ranged from <100 to <350 cells/mm^3^ except for ≥50% of the time post‐cART CD4 <100 (80% PPV for ASCUS+ and 75% for LSIL+), yielded lower or similar PPVs for identifying ASCUS+ (56%–37%) or LSIL+ (50%–26%) compared to the Cobas HPV test result alone. We observed a stepwise decrease in PPV for identifying ASCUS+ or LSIL+ when the CD4 count points ranged from <100 to <350 cells/mm^3^.

However, Cobas HPV positive test result in combination with all cut points of pre‐cART CD4, ≥50% of the time post‐cART CD4 and current CD4 had higher PPVs compared to Cobas HPV test alone for identifying ASCUS+ or LSIL+ even with a stepwise decrease in PPVs with pre‐cART CD4 count (95%–71% for ASCUS+, 94%–62% for LSIL+) and ≥50% of the time post‐cART CD4 (87%–76% for ASCUS+, 85%–68% for LSIL+) (Figure 1A and Tables S1 and S2). ≥30% of the time post‐cART CD4 alone or in combination with Cobas HPV positive test result yielded lower PPVs for ASCUS+ and LSIL+ compared to ≥50% of the time post‐cART CD4 for almost all cut points (Tables S1 and S2).

**FIGURE 2:**
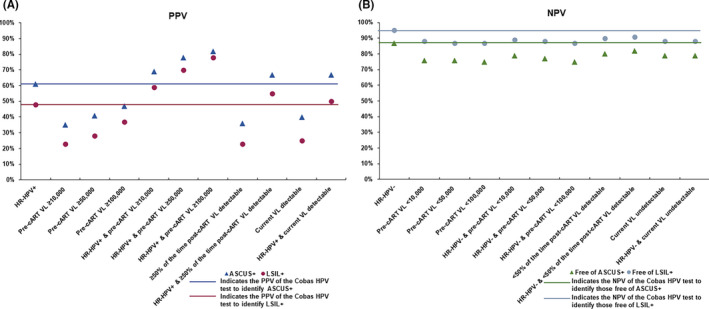
PPV (A) and NPV (B) of the Cobas HPV test alone and in combination with specific categories of pre‐cART, post‐cART, and current VL for identifying those diagnosed with or free of ASCUS+ or LSIL+

Similar to CD4 counts, we observed that all pre‐ or post‐cART or current VL measures in isolation had lower PPVs for identifying ASCUS+ or LSIL+ compared to Cobas HPV positive test result (Figure 2A and Tables S3 and S4). However, we observed that the PPVs of Cobas HPV positive test result in combination with all cut points of pre‐cART VL (82%–69% for ASCUS+, 78%–69% for LSIL+) or ≥50% of the time post‐cART detectable VL (67% for ASCUS+ and 55% for LSIL+) or current detectable VL (67% for ASCUS+ and 50% LSIL+) were significantly higher or almost similar to PPV of Cobas HPV positive test alone (Figure 2A and Tables S3 and S4). In addition, ≥30% of the time post‐cART detectable VL alone or in combination with Cobas HPV positive test result yielded slightly lower PPVs for ASCUS+ and LSIL+ compared to ≥50% of the time post‐cART VL detectable (Tables S3 and S4).

**FIGURE 3:**
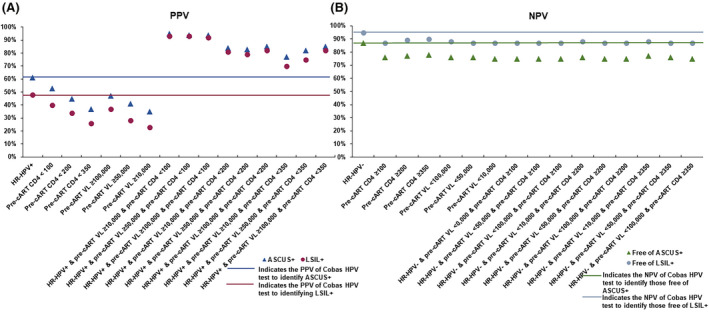
PPV (A) and NPV(B) of the Cobas HPV test in combination with pre‐cART CD4 count and pre‐cART VL for identifying those diagnosed with or free of ASCUS+ or LSIL+

PPV for identifying ASCUS+ or LSIL+ with specific combinations of all three measures, namely, Cobas HPV positive test result, CD4 count and VL measures yielded the best results. PPV for identifying ASCUS+ or LSIL+ was more than 90% when Cobas HPV test positive and pre‐cART CD4 <100 cells/mm^3^, when combined with any of the pre‐cART VL detectable categories ranging from ≥10,000 to ≥100,000 copies/mL. Similarly, PPV for identifying those diagnosed with ASCUS+ or LSIL+ was ≥79% and ≥70% when pre‐cART CD4 cut point was <200 and <350 cells/mm^3^, respectively, when pre‐cART VL ranged from ≥10,000 to ≥100,000 copies/mL (Figure 3A and Tables S5 and S6).

**FIGURE 4:**
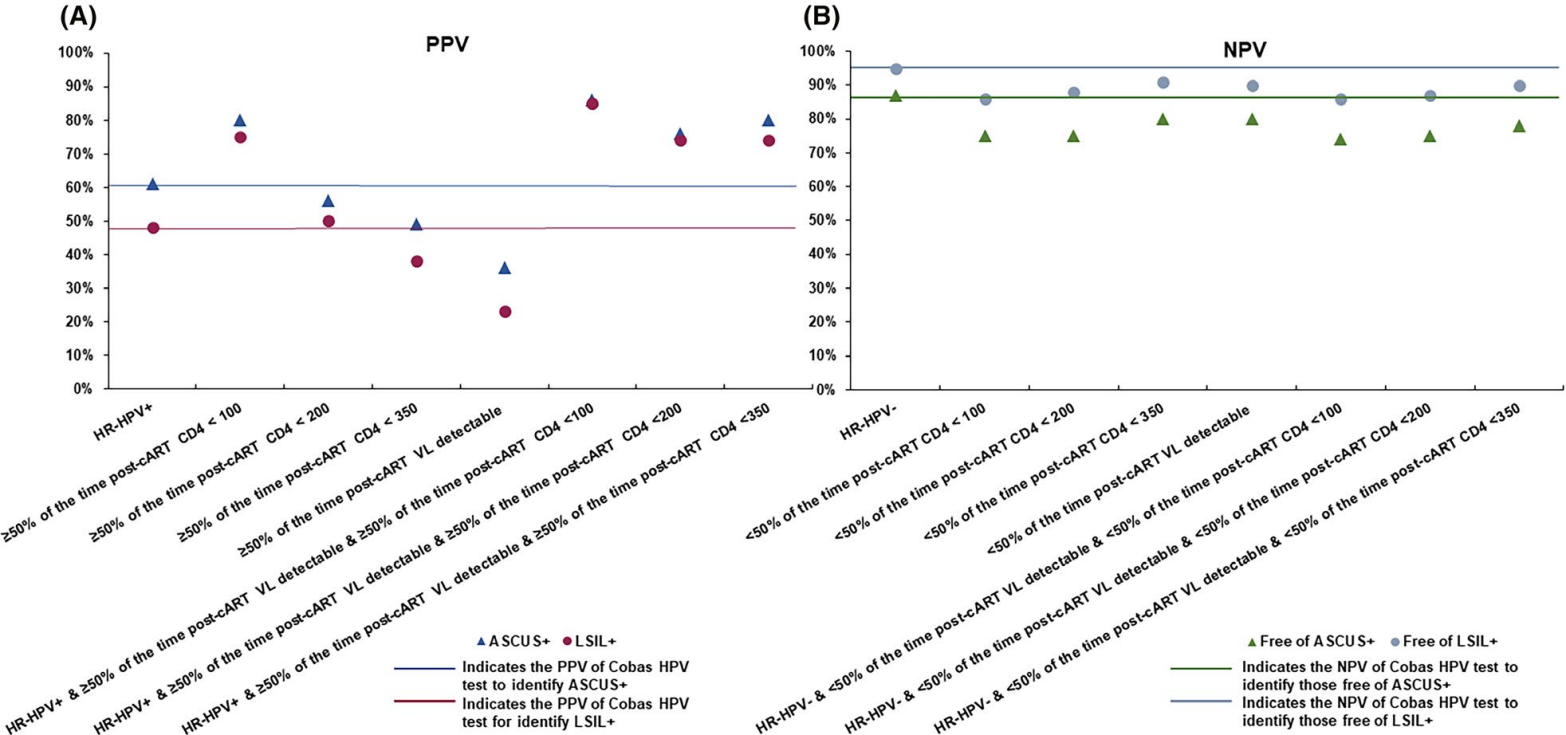
PPV (A) and NPV (B) of the Cobas HPV test in combination with post‐cART CD4 count and post‐cART VL for identifying those diagnosed with or free of ASCUS+ or LSIL+

The PPVs for identifying both ASCUS+ (80%) or LSIL+ (75%) were substantially higher than Cobas HPV positive test alone only among individuals with ≥50% of the time post‐cART CD4 <100 cells/mm^3^, but not <200 and <350 cells/mm^3^. Greater than or equal to 50% of the time post‐cART VL detectable in isolation yielded substantially lower PPV than Cobas HPV test positive result for identifying both ASCUS+ and LSIL+. However, Cobas HPV test result in combination with ≥50% of the time post‐cART CD4 cut points ranging from <100 to <350 cells/mm^3^ yielded substantially higher PPV for identifying both ASCUS+ (76%–86%) and LSIL+ (74%–85%), when combined with any of the ≥50% of the time post‐cART VL detectable categories ranging from ≥10,000 to ≥100,000 copies/mL. (Figure 4A and Tables S5 and S6). The same combination with ≥30% post‐cART VL detectable yielded slightly lower PPVs but all those values were higher than Cobas HPV positive test result alone (Tables S5 and S6).

In addition, we observed that the NPVs for identifying women free of ASCUS+ or LSIL+ with pre‐cART CD4, post‐cART CD4 (≥50% of the time), current CD4 count or VL in isolation or in combination with Cobas HPV test were consistently >70% and >85%, respectively, and therefore, NPV in some categories reached the NPV of the Cobas HPV test result alone for identifying women free of ASCUS+ (87%) or LSIL+ (95%), respectively, (Figures 1B, 2B, 3B, and 4B and Tables [Link], [Link], [Link], [Link], [Link], [Link]). In addition, ≥30% of the time post‐cART CD4 count or VL alone or in combination with Cobas HPV positive test result yielded similar NPVs for identifying women free of ASCUS+ or LSIL+ compared to ≥50% of the time post‐cART CD4 for almost all cut points (Tables [Link], [Link], [Link], [Link], [Link], [Link]).

We also assessed the PPV and NPV of the various tests for detection of ASCUS+ and LSIL+ stratified by race. CA had a higher PPV for identifying ASCUS+ (71% vs. 59%) or LSIL+ (63% vs. 44%) when positive for Cobas HPV test compared to AA. CA women also had higher PPVs for identifying ASCUS+ and LSIL+ for all pre‐/post‐cART CD4 and VL‐related variables in isolation and in combination with Cobas HPV positive test result compared to AA women. However, we observed that AA and CA women had similar NPV with Cobas HPV test for identifying women free of ASCUS+ (88% vs. 87) or LSIL+ (95% vs. 97%). AA women also had similar or slightly higher NPVs for identifying women free of ASCUS+ or LSIL+ for all pre‐/post‐cART CD4 and VL‐related variables in isolation and in combination with Cobas HPV negative test result compared to CA women (data not shown).

## 6 DISCUSSION

HPV DNA detection has been widely incorporated into CC prevention programs since HPV tests are biochemical, readily standardized and have higher NPV for identifying women without precancerous lesions compared to cytology tests that are subjective and require visual identification by experienced pathologists.^16^ However, current HPV DNA tests do not provide a desired PPV for identification of women with precancerous lesions. This is of concern, as targeted referrals of women with precancerous lesions for further care will play a major role in reducing the risk of developing this most preventable cancer, especially in many regions of the world with high rates of CC due to limited resource settings to implement other preventive measures. Studies have suggested the need for advanced technology such as next‐generation sequencing of the HPV genome and development of tests based on genetic and/or epigenetic changes and other molecular biomarkers to improve the PPV of HPV based tests.^17^ However, application of these novel tests could be a challenge in many clinical settings because of the amount of information generated, its complexity and cost. We report the possibility of improving the PPV for identifying high‐risk women by using a widely available and FDA approved HPV test in combination with HIV‐related clinical data, also available in routine care settings of many HIV care settings.

As expected, we observed that the NPV of the Cobas HPV test was highly satisfactory for identifying women without abnormal cytology, especially LSIL+ irrespective of race. However, the PPV value of the Cobas HPV test for identifying LSIL+ was only 48% in the overall population and 44% in AA women. Our observation that most of the pre‐cART, post‐cART, and current CD4 cut points and VL measures in isolation yielded lower or similar PPVs for identifying ASCUS+ or LSIL+ compared to Cobas HPV test result alone indicated that these HIV clinical variables in isolation are not useful for identifying those with underlying lesions. In contrast, NPV of the same variables in isolation reached the NPV of the Cobas HPV test result alone, especially for excluding the presence of LSIL+ indicating their usefulness in identifying women with no disease. This finding provides a tool for identifying low risk women in a clinical setting where HIV‐related variables are available but not equipped to do HPV testing.

More importantly, Cobas HPV test in combination with all cut points of pre‐/post‐cART and current CD4 counts had higher PPVs compared to Cobas HPV test alone for identifying ASCUS+ or LSIL+, yielding higher than 90% PPV with some cut points. We noted that women with pre‐cART CD4 counts <100 in combination with HR‐HPV positive status had the highest PPV of 95% and 94% for identifying women diagnosed with ASCUS+ or LSIL+, respectively. It is known that HIV+ receiving cART can partially restore immune functions. However, persistent systemic inflammation driving their immunodeficiency may persist, particularly in people initiating ART at lower CD4 counts^18^, ^19^, ^20^ and this may also result in the persistence of HR‐HPVs in the cervix that cause precancerous lesions. This phenomenon supports that CD4 status may facilitate HPV carcinogenesis, and therefore, contributes to a higher PPV to identify women with ASCUS+. Therefore, utilization of CD4 values in combination with any abnormal pap is a useful and a logical approach to identify women at high risk for developing CC with higher accuracy.

Even though the PPV of Cobas HPV test in combination with VL load was higher than the Cobas HPV test alone, those were somewhat lower than for CD4‐Cobas HPV combinations, suggesting that CD4 counts are more informative than VL in identifying women at risk. Cobas HPV test in combination with all cut points of pre‐cART, post‐cART, and current CD4 counts and VL yielded the best PPVs, especially, >90% PPV with some categories of pre‐cART variables, indicating the importance of knowing patients CD4, VL status during pre‐cART in identifying their risk of developing CC. Overall, our results also suggested that variation in VL from ≥10,000 to ≥100,000 copies/mL had no significant effect on PPV emphasizing the fact that CD4 counts are more informative than VL in identifying women at high risk. Our results also indicated that Cobas HPV test alone or in combination of pre‐cART, post‐cART, and current CD4 counts and VL measures at all cut points are likely to be more useful in identifying abnormal cytology among CA women than AA women suggesting the need to explore other markers that will be beneficial for identifying abnormal cytology in this ethnic group.

Our results may also apply to women who had been given HPV vaccines or with effective adherence to cART for the following reasons; Even though HPV vaccines mainly target young adolescents, vaccines are also recommended up through 26 years of age in immunocompromised individuals, including those infected with HIV despite that fact they may get only partial benefit because of their exposure to HPV and its entry into disrupted mucosal epithelium facilitated by HIV tat and gp120 proteins prior to vaccination.^21^, ^22^ However, it is likely that our population is exposed to HPV vaccines and has at least partial benefit on HPV infections/cervical lesions. Lower prevalence HPV genotypes 16/18 and higher‐grade lesions in our study population is suggestive of some women's exposure to HPV vaccines. In vaccinated women, the accuracy of HPV testing alone for identifying women at high risk for developing CC may change as infections with HPV vaccine genotypes are reduced with effective vaccine uptake.^23^ Previous studies that evaluated the relationship between cART initiation, high‐risk HPV, and HPV‐related cervical lesions in WWH have shown that women using cART had a lower prevalence of high‐risk HPV infections or abnormal cervical cytology than women who were not using cART.^24^ Lower prevalence HPV genotypes and higher‐grade lesions in our population may reflect their adherence to cART. Collectively, lower PPV of Cobas HPV test alone for identifying ASCUS+ and LSIL+ is reflective of these possibilities. Therefore, our results provide a more accurate tool for managing vaccinated women or those taking cART by combining Cobas HPV results with CD4 and VL, especially those who had an undesirable CD4 and VL status during the pre‐cART period.

Our study has several strengths including a population representative of WWH receiving care in the Deep South, which is disproportionately impacted by the HIV epidemic in the United States; a good assessment of cervical cytology, HPV status, and the availability of CD4 and VL results over a long‐time period. A limitation of the study is using cytology and HPV results at only one time point. Gathering follow‐up data on these variables and using a summary measure of those and confirmation of histological diagnoses of lesions by targeted referral of women for colposcopy based on our results will strengthen the findings of this study. Replication of these findings in other populations of WWH will increase the scientific credibility and application of our findings in the management of WWH at risk for developing CC.

## CONFLICT OF INTEREST

None.

## ETHICAL STATEMENT

The UAB Institutional Review Board for Protection of Human Subjects approved the study.

## Supporting information

Table S1

Table S2

Table S3

Table S4

Table S5

Table S6

## Data Availability

The data that support the findings of this study are available from the corresponding author upon reasonable request.
