# Supplementary material for: The accuracy of HPV genotyping in isolation and in combination with CD4 and HIV viral load for the identification of HIV‐infected women at risk for developing cervical cancer
Source: Cancer Med. 2021 Feb 19;10(5):1900–9. doi: 10.1002/cam4.3785 (PMC7940247; doi:10.1002/cam4.3785)
Supplement: Supplementary file 1 — Table S1 [file CAM4-10-1900-s002.docx]

**Supplementary Table 1.** PPV and NPV of Cobas HPV test alone and in combination with specific categories of pre-cART, post-cART and current CD4 count for identifying ASCUS+, n=98 or NILM, n=246

| **Test** | **TP^a^** | **TN^b^** | **FP^c^** | **FN^d^** | **PPV^e^** | **NPV^f^** |
| --- | --- | --- | --- | --- | --- | --- |
| Cobas HPV test | 69 | 202 | 44 | 29 | 61%  (53.8%-67.8%) | 87%  (83.6%-90.5%) |
| Pre-cART CD4 <100 cells/mm^3^ | 30 | 219 | 27 | 68 | 53%  (41.1%-63.9%) | 76%  (73.7%-78.7%) |
| Pre-cART CD4 <200 cells/mm^3^ | 41 | 195 | 51 | 57 | 45%  (36.4%-53.0%) | 77%  (74.1%-80.4%) |
| Pre-cART CD4 < 350 cells/mm^3^ | 55 | 154 | 92 | 43 | 37%  (32.0%-43.1%) | 78%  (73.7%-82.0%) |
| ≥50% of the time post-cART CD4 < 100 cells/mm^3^ | 16 | 242 | 4 | 82 | 80%  (57.8%-92.1%) | 75%  (73.0%-76.3%) |
| ≥30% of the time post-cART CD4 < 100 cells/mm^3^ | 17 | 231 | 15 | 81 | 53%  (37.1%-68.5%) | 74%  (72.1%-75.8%) |
| ≥50% of the time post-cART CD4 < 200 cells/mm^3^ | 23 | 228 | 18 | 75 | 56%  (41.9%-69.3%) | 75%  (73.0%-77.3%) |
| ≥30% of the time post-cART CD4 < 200 cells/mm^3^ | 37 | 209 | 37 | 61 | 50%  (40.3%-59.7%) | 77%  (74.4%-80.1%) |
| ≥50% of the time post-cART CD4 <350 cells/mm^3^ | 50 | 193 | 53 | 48 | 49%  (41.0%-56.2%) | 80%  (76.5%-83.3%) |
| ≥30% of the time post-cART CD4 <350 cells/mm^3^ | 61 | 168 | 78 | 37 | 44%  (38.1%-49.8%) | 82%  (77.6%-85.6%) |
| Current CD4 <100 cells/mm^3^ | 13 | 240 | 6 | 85 | 68%  (45.9%-84.7%) | 74%  (72.3%-75.4%) |
| Current CD4 <200 cells/mm^3^ | 18 | 229 | 17 | 80 | 51%  (36.3%-66.3%) | 74%  (72.1%-76.0%) |
| Current CD4 <350 cells/mm^3^ | 34 | 208 | 38 | 64 | 47%  (37.5%-57.2%) | 76%  (73.6%-79.1%) |
| Cobas HPV test & pre-cART CD4 <100 cells/mm^3^ | 20 | 245 | 1 | 78 | 95%  (73.1%-99.3%) | 76%  (74.0%-77.6%) |
| Cobas HPV test & pre-cART CD4 <200 cells/mm^3^ | 27 | 239 | 7 | 71 | 79%  (63.5%-89.5%) | 77%  (74.8%-79.2%) |
| Cobas HPV test & pre-cART CD4 <350 cells/mm^3^ | 36 | 231 | 15 | 62 | 71%  (57.9%-80.7%) | 79%  (76.2%-81.3%) |
| Cobas HPV test & ≥50% of the time post-cART CD4 < 100 | 13 | 244 | 2 | 85 | 87%  (59.9%-96.6%) | 74%  (72.6%-75.6%) |
| Cobas & ≥30% of the time post-cART CD4 < 100 | 13 | 242 | 4 | 85 | 76%  (52.1%-90.7%) | 74%  (72.5%-75.5%) |
| Cobas HPV test & ≥50% of the time post-cART CD4 < 200 | 17 | 241 | 5 | 81 | 77%  (56.3%-90.0%) | 75%  (73.1%-76.5%) |
| Cobas HPV test & ≥30% of the time post-cART CD4 < 200 | 28 | 238 | 8 | 70 | 78%  (62.3%-88.1%) | 77%  (75.0%-79.4%) |
| Cobas HPV test & ≥50% of the time post-cART CD4 <350 | 39 | 234 | 12 | 59 | 76%  (64.0%-85.6%) | 80%  (77.1%-82.4%) |
| Cobas HPV test & ≥30% of the time post-cART CD4 <350 | 46 | 228 | 18 | 52 | 72%  (61.0%-80.7%) | 81%  (78.4%-84.1%) |
| Cobas HPV test & current CD4 <100 cells/mm^3^ | 9 | 244 | 2 | 89 | 82%  (49.7%-95.3%) | 73%  (72.0%-74.5%) |
| Cobas HPV test & current CD4 <200 cells/mm^3^ | 14 | 240 | 6 | 84 | 70%  (48.0%-85.5%) | 74%  (72.4%-75.6%) |
| Cobas HPV test & current CD4 <350 cells/mm^3^ | 29 | 237 | 9 | 69 | 76%  (61.3%-86.8%) | 77%  (75.1%-79.7%) |

^a^ TP- true positive , ^b^ TN- true negative ^c^ FN- false negative, ^d^ FP- false positive, ^e^ PPV- positive predictive value, ^f^ NPV- negative predictive value
