# Supplementary material for: The accuracy of HPV genotyping in isolation and in combination with CD4 and HIV viral load for the identification of HIV‐infected women at risk for developing cervical cancer
Source: Cancer Med. 2021 Feb 19;10(5):1900–9. doi: 10.1002/cam4.3785 (PMC7940247; doi:10.1002/cam4.3785)
Supplement: Supplementary file 2 — Table S2 [file CAM4-10-1900-s004.docx]

**Supplementary Table 2.** PPV and NPV of Cobas HPV test alone and in combination with specific categories of pre-cART, post-cART and current CD4 count for identifying LSIL+ n=50 or NILM, n=246

| **Test** | **TP^a^** | **TN^b^** | **FP^c^** | **FN^d^** | **PPV^e^** | **NPV^f^** |
| --- | --- | --- | --- | --- | --- | --- |
| Cobas HPV test | 40 | 202 | 44 | 10 | 48%  (40.2%-55.1%) | 95%  (92.0%-97.2%) |
| Pre-cART CD4 <100 cells/mm^3^ | 18 | 219 | 27 | 32 | 40%  (28.5%-52.7%) | 87%  (84.7%-89.4%) |
| Pre-cART CD4 < 200 cells/mm^3^ | 26 | 195 | 51 | 24 | 34%  (26.2%-42.3%) | 89%  (85.8%-91.6%) |
| Pre-cART CD4 < 350 cells/mm^3^ | 32 | 154 | 92 | 18 | 26%  (21.1%-31.2%) | 90%  (85.4%-92.6%) |
| ≥50% of the time post-cART CD4 <100 cells/mm^3^ | 12 | 242 | 4 | 38 | 75%  (50.2%-89.9%) | 86%  (84.5%-88.2%) |
| ≥30% of the time post-cART CD4 <100 cells/mm^3^ | 13 | 231 | 15 | 37 | 46%  (30.6%-63.1%) | 86%  (84.1%-88.1%) |
| ≥50% of the time post-cART CD4 <200 cells/mm^3^ | 18 | 228 | 18 | 32 | 50%  (35.9%-64.1%) | 88%  (85.2%-89.8%) |
| ≥30% of the time post-cART CD4 <200 cells/mm^3^ | 24 | 209 | 37 | 26 | 39%  (30.0%-49.5%) | 89%  (86.0%-91.3%) |
| ≥50% of the time post-cART CD4 <350 cells/mm^3^ | 32 | 193 | 53 | 18 | 38%  (30.6%-45.3%) | 91%  (88.0%-94.0%) |
| ≥30% of the time post-cART CD4 <350 cells/mm^3^ | 35 | 168 | 78 | 15 | 31%  (25.7%-36.7%) | 92%  (87.9%-94.5%) |
| Current CD4 <100 cells/mm^3^ | 10 | 240 | 6 | 40 | 63%  (38.8%-81.4%) | 86%  (83.9%-87.3%) |
| Current CD4 <200 cells/mm^3^ | 13 | 229 | 17 | 37 | 43%  (28.4%-59.5%) | 86%  (84.0%-88.0%) |
| Current CD4 <350 cells/mm^3^ | 21 | 208 | 38 | 29 | 36%  (26.3%-46.1%) | 88%  (84.9%-90.1%) |
| Cobas HPV test & pre-cART CD4 <100 cells/mm^3^ | 15 | 245 | 1 | 35 | 94%  (67.0%-99.1%) | 88%  (85.4%-89.4%) |
| Cobas HPV test & pre-cART CD4 <200 cells/mm^3^ | 20 | 239 | 7 | 30 | 74%  (56.1%-86.5%) | 89%  (86.4%-90.9%) |
| Cobas HPV test & pre-cART CD4 <350 cells/mm^3^ | 24 | 231 | 15 | 26 | 62%  (47.5%-73.9%) | 90%  (87.2%-92.1%) |
| Cobas HPV test & ≥50% of the time post-cART CD4 <100 cells/mm^3^ | 11 | 244 | 2 | 39 | 85%  (55.7%-96.0%) | 86%  (84.4%-87.9%) |
| Cobas HPV test & ≥30% of the time post-cART CD4 <100 cells/mm^3^ | 11 | 242 | 4 | 39 | 73%  (47.7%-89.2%) | 86%  (84.3%-87.8%) |
| Cobas HPV test & ≥50% of the time post-cART CD4 < 200 cells/mm^3^ | 14 | 241 | 5 | 36 | 74%  (51.4%-88.1%) | 87%  (84.9%-88.8%) |
| Cobas HPV test & ≥30% of the time post-cART CD4 < 200 cells/mm^3^ | 20 | 238 | 8 | 30 | 71%  (53.9%-84.3%) | 89%  (86.3%-90.9%) |
| Cobas HPV test & ≥50% of the time post-cART CD4 <350 cells/mm^3^ | 26 | 234 | 12 | 24 | 68%  (54.0%-80.0%) | 91%  (87.9%-92.9%) |
| Cobas HPV test & ≥30% of the time post-cART CD4 <350 cells/mm^3^ | 28 | 228 | 18 | 22 | 61%  (48.3%-72.1%) | 91%  (88.3%-93.4%) |
| Cobas HPV test & current CD4 <100 cells/mm^3^ | 7 | 244 | 2 | 43 | 78%  (42.8%-94.2%) | 85%  (83.5%-86.4%) |
| Cobas HPV test & current CD4 <200 cells/mm^3^ | 10 | 240 | 6 | 40 | 63%  (38.8%-81.4%) | 86%  (83.9%-87.3%) |
| Cobas HPV test & current CD4 <350 cells/mm^3^ | 18 | 237 | 9 | 32 | 67%  (48.8%-80.7%) | 88%  (85.7%-90.1%) |

^a^ TP- true positive , ^b^ TN- true negative ^c^ FN- false negative, ^d^ FP- false positive, ^e^ PPV- positive predictive value, ^f^ NPV- negative predictive value
