# Supplementary material for: The accuracy of HPV genotyping in isolation and in combination with CD4 and HIV viral load for the identification of HIV‐infected women at risk for developing cervical cancer
Source: Cancer Med. 2021 Feb 19;10(5):1900–9. doi: 10.1002/cam4.3785 (PMC7940247; doi:10.1002/cam4.3785)
Supplement: Supplementary file 3 — Table S3 [file CAM4-10-1900-s006.docx]

**Supplementary Table 3.** PPV and NPV of Cobas HPV test alone and in combination with pre-cART, post-cART and current VL measure for identifying ASCUS+, n=98 or NILM, n=246

| **Test** | **TP^a^** | **TN^b^** | **FP^c^** | **FN^d^** | **PPV^e^** | **NPV^f^** |
| --- | --- | --- | --- | --- | --- | --- |
| Cobas HPV test | 69 | 202 | 44 | 29 | 61%  (53.8%-67.8%) | 87%  (83.6%-90.5%) |
| Pre-cART VL ≥ 10,000 copies/mL | 52 | 149 | 97 | 46 | 35%  (29.6%-40.6%) | 76%  (71.9%-80.4%) |
| Pre-cART VL ≥ 50,000 copies/mL | 36 | 195 | 51 | 62 | 41%  (33.1%-50.2%) | 76%  (72.8%-78.7%) |
| Pre-cART VL ≥ 100,000 copies/mL | 28 | 215 | 31 | 70 | 47%  (36.4%-58.7%) | 75%  (72.9%-77.8%) |
| ≥50% of the time post-cART VL detectable | 66 | 130 | 116 | 32 | 36%  (32.0%-40.8%) | 80%  (74.9%-84.7%) |
| ≥30% of the time post-cART VL detectable | 78 | 89 | 157 | 20 | 33%  (30.2%-36.3%) | 82%  (74.4%-87.2%) |
| Current VL detectable | 54 | 166 | 80 | 44 | 40%  (34.4%-46.5%) | 79%  (74.9%-82.7%) |
| Cobas HPV test & pre-cART VL ≥ 10,000 copies/mL | 35 | 230 | 16 | 63 | 69%  (56.0%-79.0%) | 79%  (75.8%-80.9%) |
| Cobas HPV test & pre-cART VL ≥ 50,000 copies/mL | 25 | 239 | 7 | 73 | 78%  (61.5%-88.9%) | 77%  (74.4%-78.6%) |
| Cobas HPV test & pre-cART VL ≥ 100,000 copies/mL | 18 | 242 | 4 | 80 | 82%  (61.0%-92.8%) | 75%  (73.3%-76.9%) |
| Cobas HPV test & ≥50% of the time post-cART VL detectable | 49 | 222 | 24 | 49 | 67%  (57.1%-75.8%) | 82%  (78.7%-84.7%) |
| Cobas HPV test & ≥30% of the time Post-cART VL detectable | 56 | 215 | 31 | 42 | 64%  (55.5%-72.4%) | 84%  (80.2%-86.6%) |
| Cobas HPV test & current VL detectable | 36 | 228 | 18 | 62 | 67%  (54.4%-77.0%) | 79%  (75.9%-81.1%) |

^a^ TP- true positive , ^b^ TN- true negative ^c^ FN- false negative, ^d^ FP- false positive, ^e^ PPV- positive predictive value, ^f^ NPV- negative predictive value
