# Supplementary material for: The accuracy of HPV genotyping in isolation and in combination with CD4 and HIV viral load for the identification of HIV‐infected women at risk for developing cervical cancer
Source: Cancer Med. 2021 Feb 19;10(5):1900–9. doi: 10.1002/cam4.3785 (PMC7940247; doi:10.1002/cam4.3785)
Supplement: Supplementary file 4 — Table S4 [file CAM4-10-1900-s003.docx]

**Supplementary Table 4.** PPV and NPV of Cobas HPV test alone and in combination with specific categories of pre-cART, post-cART and current VL measure for identifying LSIL+, n=50 or NILM, n=246

| **Test** | **TP^a^** | **TN^b^** | **FP^c^** | **FN^d^** | **PPV^e^** | **NPV^f^** |
| --- | --- | --- | --- | --- | --- | --- |
| Cobas HPV test | 40 | 202 | 44 | 10 | 48%  (40.2%-55.1%) | 95%  (92.0%-97.2%) |
| Pre-cART VL ≥ 10,000 copies/mL | 29 | 149 | 97 | 21 | 23%  (18.4%-28.4%) | 88%  (83.5%-90.9%) |
| Pre-cART VL ≥ 50,000 copies/mL | 20 | 195 | 51 | 30 | 28%  (20.5%-37.3%) | 87%  (83.7%-89.2%) |
| Pre-cART VL ≥ 100,000 copies/mL | 18 | 215 | 31 | 32 | 37%  (26.1%-48.8%) | 87%  (84.4%-89.3%) |
| ≥50% of the time post-cART VL detectable | 35 | 130 | 116 | 15 | 23%  (19.4%-27.4%) | 90%  (84.8%-93.1%) |
| ≥30% of the time post-cART VL detectable | 40 | 89 | 157 | 10 | 20%  (17.7%-23.2%) | 90%  (83.3%-94.1%) |
| Current VL detectable | 27 | 166 | 80 | 23 | 25%  (19.8%-31.6%) | 88%  (84.1%-90.8%) |
| Cobas HPV test & pre-cART VL ≥ 10,000 copies/mL | 23 | 230 | 16 | 27 | 59%  (45.1%-71.6%) | 89%  (86.8%-91.7%) |
| Cobas HPV test & pre-cART VL ≥ 50,000 copies/mL | 16 | 239 | 7 | 34 | 70%  (49.8%-84.0%) | 88%  (85.3%-89.5%) |
| Cobas HPV tes & pre-cART VL ≥ 100,000 copies/mL | 14 | 242 | 4 | 36 | 78%  (54.6%-91.1%) | 87%  (85.0%-88.9%) |
| Cobas HPV test & ≥50% of the time post-cART VL detectable | 29 | 222 | 24 | 21 | 55%  (43.6%-65.4%) | 91%  (88.4%-93.6%) |
| Cobas HPV test & ≥30% of the time post-cART VL detectable | 33 | 215 | 31 | 17 | 52%  (42.0%-61.0%) | 93%  (89.6%-94.9%) |
| Cobas HPV test & current VL detectable | 18 | 228 | 18 | 32 | 50%  (35.9%-64.1%) | 88%  (85.2%-89.8%) |

^a^ TP- true positive , ^b^ TN- true negative ^c^ FN- false negative, ^d^ FP- false positive, ^e^ PPV- positive predictive value, ^f^ NPV- negative predictive value
