# Supplementary material for: The accuracy of HPV genotyping in isolation and in combination with CD4 and HIV viral load for the identification of HIV‐infected women at risk for developing cervical cancer
Source: Cancer Med. 2021 Feb 19;10(5):1900–9. doi: 10.1002/cam4.3785 (PMC7940247; doi:10.1002/cam4.3785)
Supplement: Supplementary file 5 — Table S5 [file CAM4-10-1900-s005.docx]

**Supplementary Table 5**. PPV and NPV for the combination Cobas HPV test, pre-cART and post-cART CD4 count and VL measure for identifying ASCUS+, n=98 or NILM, n=246

| **Test** | **TP^a^** | **TN^b^** | **FP^c^** | **FN^d^** | **PPV^e^** | **NPV^f^** |
| --- | --- | --- | --- | --- | --- | --- |
| Cobas HPV test & pre-cART VL ≥ 10,000 copies/mL & pre-cART CD4 <100 cells/mm^3^ | 18 | 245 | 1 | 80 | 95%  (70.9%-99.3%) | 75%  (73.6%-77.1%) |
| Cobas HPV test & pre-cART VL ≥ 50,000 copies/mL & pre-cART CD4 <100 cells/mm^3^ | 17 | 245 | 1 | 81 | 94%  (69.6%-99.2%) | 75%  (73.4%-76.8%) |
| Cobas HPV test & pre-cART VL ≥ 100,000 copies/mL & pre-cART CD4<100 cells/mm^3^ | 15 | 245 | 1 | 83 | 94%  (66.8%-99.1%) | 75%  (73.1%-76.3%) |
| Cobas HPV test & pre-cART VL ≥ 10,000 copies/mL & pre-cART CD4 <200 cells/mm^3^ | 21 | 242 | 4 | 77 | 84%  (64.9%-93.7%) | 76%  (73.9%-77.7%) |
| Cobas HPV test & pre-cART VL ≥ 50,000 copies/mL & pre-cART CD4 <200 cells/mm^3^ | 19 | 242 | 4 | 79 | 83%  (62.4%-93.2%) | 75%  (73.5%-77.2%) |
| Cobas HPV test & pre-cART VL ≥ 100,000 copies/mL & pre-cART CD4 <200 cells/mm^3^ | 17 | 243 | 3 | 81 | 85%  (62.9%-95.0%) | 75%  (73.2%-76.7%) |
| Cobas HPV test & pre-cART VL ≥ 10,000 copies/mL & pre-cART CD4 <350 cells/mm^3^ | 27 | 238 | 8 | 71 | 77%  (61.4%-87.8%) | 77%  (74.8%-79.1%) |
| Cobas HPV test & pre-cART VL ≥ 50,000 copies/mL & pre-cART CD4 <350 cells/mm^3^ | 23 | 241 | 5 | 75 | 82%  (64.3%-92.2%) | 76%  (74.2%-78.2%) |
| Cobas HPV test & pre-cART VL ≥ 100,000 copies/mL & pre-cART CD4 <350 cells/mm^3^ | 17 | 243 | 3 | 81 | 85%  (62.9%-95.0%) | 75%  (73.2%-76.7%) |
| Cobas HPV test & ≥50% of the time post-cART VL detectable & >50% of the time post-cART CD4 <100 cells/mm^3^ | 12 | 244 | 2 | 86 | 86%  (57.8%-96.3%) | 74%  (72.5%-75.4%) |
| Cobas HPV test & ≥50% of the time post-cART VL detectable & ≥50% of the time post-cART CD4 <200 cells/mm^3^ | 16 | 241 | 5 | 82 | 76%  (54.7%-89.5%) | 75%  (72.9%-76.3%) |
| HR-HPV+ & ≥50% of the time post-cART VL detectable & ≥50% of the time post-cART CD4 <350 cells/mm^3^ | 32 | 238 | 8 | 66 | 80%  (65.7%-89.3%) | 78%  (75.8%-80.6%) |
| Cobas HPV test & ≥50% of the time post-cART VL detectable & ≥30% of the time post-cART CD4 <100 cells/mm^3^ | 12 | 242 | 4 | 86 | 75%  (49.8%-90.1%) | 74%  (72.3%-75.2%) |
| Cobas HPV test & ≥50% of the time post-cART VL detectable & ≥30% of the time post-cART CD4 <200 cells/mm^3^ | 22 | 239 | 7 | 76 | 76%  (58.1%-87.7%) | 76%  (73.8%-77.8%) |
| Cobas HPV test & ≥50% of the time post-cART VL detectable & ≥30% of the time post-cART CD4 <350 cells/mm^3^ | 38 | 233 | 13 | 60 | 75%  (62.0%-84.0%) | 80%  (76.8%-82.0%) |
| Cobas HPV test & ≥30% of the time post-cART VL detectable & ≥50% of the time post-cART CD4 <100 cells/mm^3^ | 12 | 244 | 2 | 86 | 86%  (57.8%-96.3%) | 74%  (72.5%-75.4%) |
| Cobas HPV test & ≥30% of the time post-cART VL detectable & ≥50% of the time post-cART CD4 <200 cells/mm^3^ | 16 | 241 | 5 | 82 | 76%  (54.7%-89.5%) | 75%  (72.9%-76.3%) |
| Cobas HPV test & ≥30% of the time post-cART VL detectable & ≥50% of the time post-cART CD4 <350 cells/mm^3^ | 36 | 237 | 9 | 62 | 80%  (66.7%-88.9%) | 79%  (76.6%-81.7%) |
| Cobas HPV test & ≥30% of the time post-cART VL detectable & ≥30% of the time post-cART CD4 <100 cells/mm^3^ | 12 | 242 | 4 | 86 | 75%  (49.8%-90.1%) | 74%  (72.3%-75.2%) |
| Cobas HPV test & ≥30% of the time post-cART VL detectable & ≥30% of the time post-cART CD4 <200 cells/mm^3^ | 25 | 239 | 7 | 73 | 78%  (61.5%-88.9%) | 77%  (74.4%-78.6%) |
| Cobas HPV test & ≥30% of the time post-cART VL detectable & ≥30% of the time post-cART CD4 <350 cells/mm^3^ | 43 | 232 | 14 | 55 | 75%  (63.8%-84.3%) | 80%  (77.9%-83.4%) |

^a^ TP- true positive , ^b^ TN- true negative ^c^ FN- false negative, ^d^ FP- false positive, ^e^ PPV- positive predictive value, ^f^ NPV- negative predictive value
