# Supplementary material for: The accuracy of HPV genotyping in isolation and in combination with CD4 and HIV viral load for the identification of HIV‐infected women at risk for developing cervical cancer
Source: Cancer Med. 2021 Feb 19;10(5):1900–9. doi: 10.1002/cam4.3785 (PMC7940247; doi:10.1002/cam4.3785)
Supplement: Supplementary file 6 — Table S6 [file CAM4-10-1900-s001.docx]

**Supplementary Table 6**. PPV and NPV for the combination Cobas HPV test, pre-cART and post-cART CD4 count and VL measure for identifying LSIL+, n=50 or NILM, n=246

| **Test** | **TP^a^** | **TN^b^** | **FP^c^** | **FN^d^** | **PPV^e^** | **NPV^f^** |
| --- | --- | --- | --- | --- | --- | --- |
| Cobas HPV test & pre-cART VL ≥ 10,000 copies/mL & pre-cART CD4 <100 cells/mm^3^ | 14 | 245 | 1 | 36 | 93%  (65.3%-99.0%) | 87%  (85.1%-89.0%) |
| Cobas HPV test & pre-cART VL ≥ 50,000 copies/mL & pre-cART CD4 <100 cells/mm^3^ | 13 | 245 | 1 | 37 | 93%  (63.5%-99.0%) | 87%  (84.9%-88.6%) |
| Cobas HPV test & pre-cART VL ≥ 100,000 copies/mL & pre-cART CD4<100 cells/mm^3^ | 12 | 245 | 1 | 38 | 92%  (61.5%-98.9%) | 87%  (84.7%-88.3%) |
| Cobas HPV test & pre-cART VL ≥ 10,000 copies/mL & pre-cART CD4 <200 cells/mm^3^ | 17 | 242 | 4 | 33 | 81%  (59.9%-92.4%) | 88%  (85.7%-90.0%) |
| Cobas HPV test & pre-cART VL ≥ 50,000 copies/mL & pre-cART CD4 <200 cells/mm^3^ | 15 | 242 | 4 | 35 | 79%  (56.5%-91.5%) | 87%  (85.2%-89.2%) |
| Cobas HPV test & pre-cART VL ≥ 100,000 copies/mL & pre-cART CD4 <200 cells/mm^3^ | 14 | 243 | 3 | 36 | 82%  (58.2%-94.0%) | 87%  (85.0%-88.9%) |
| Cobas HPV test & pre-cART VL ≥ 10,000 copies/mL & pre-cART CD4 <350 cells/mm^3^ | 19 | 238 | 8 | 31 | 70%  (52.4%-83.7%) | 88%  (86.1%-90.5%) |
| Cobas HPV test & pre-cART VL ≥ 50,000 copies/mL & pre-cART CD4 <350 cells/mm^3^ | 15 | 241 | 5 | 35 | 75%  (53.3%-88.7%) | 87%  (85.2%-89.2%) |
| Cobas HPV test & pre-cART VL ≥ 100,000 copies/mL & pre-cART CD4 <350 cells/mm^3^ | 14 | 243 | 3 | 36 | 82%  (58.2%-94.0%) | 87%  (85.0%-88.9%) |
| Cobas HPV test & ≥50% of the time post-cART VL detectable & >50% of the time post-cART CD4 <100 cells/mm^3^ | 11 | 244 | 2 | 39 | 85%  (55.7%-96.0%) | 86%  (84.4%-87.9%) |
| Cobas HPV test & ≥50% of the time post-cART VL detectable & ≥50% of the time post-cART CD4 <200 cells/mm^3^ | 14 | 241 | 5 | 36 | 74%  (51.4%-88.1%) | 87%  (84.9%-88.8%) |
| HR-HPV+ & ≥50% of the time post-cART VL detectable & ≥50% of the time post-cART CD4 <350 cells/mm^3^ | 23 | 238 | 8 | 27 | 74%  (57.7%-85.8%) | 90%  (87.2%-91.9%) |
| Cobas HPV test & ≥50% of the time post-cART VL detectable & ≥30% of the time post-cART CD4 <100 cells/mm^3^ | 11 | 242 | 4 | 39 | 73%  (47.7%-89.2%) | 86%  (84.3%-87.8%) |
| Cobas HPV test & ≥50% of the time post-cART VL detectable & ≥30% of the time post-cART CD4 <200 cells/mm^3^ | 18 | 239 | 7 | 32 | 72%  (53.1%-85.4%) | 88%  (85.8%-90.2%) |
| Cobas HPV test & ≥50% of the time post-cART VL detectable & ≥30% of the time post-cART CD4 <350 cells/mm^3^ | 25 | 233 | 13 | 25 | 66%  (51.4%-77.8%) | 90%  (87.6%-92.5%) |
| Cobas HPV test & ≥30% of the time post-cART VL detectable & ≥50% of the time post-cART CD4 <100 cells/mm^3^ | 11 | 244 | 2 | 39 | 85%  (55.7%-96.0%) | 86%  (84.4%-87.9%) |
| Cobas HPV test & ≥30% of the time post-cART VL detectable & ≥50% of the time post-cART CD4 <200 cells/mm^3^ | 14 | 241 | 5 | 36 | 74%  (51.4%-88.1%) | 87%  (84.9%-88.8%) |
| Cobas HPV test & ≥30% of the time post-cART VL detectable & ≥50% of the time post-cART CD4 <350 cells/mm^3^ | 26 | 237 | 9 | 24 | 74%  (59.1%-85.3%) | 91%  (88.1%-93.0%) |
| Cobas HPV test & ≥30% of the time post-cART VL detectable & ≥30% of the time post-cART CD4 <100 cells/mm^3^ | 11 | 242 | 4 | 39 | 73%  (47.7%-89.2%) | 86%  (84.3%-87.8%) |
| Cobas HPV test & ≥30% of the time post-cART VL detectable & ≥30% of the time post-cART CD4 <200 cells/mm^3^ | 20 | 239 | 7 | 30 | 74%  (56.1%-86.5%) | 89%  (86.4%-90.9%) |
| Cobas HPV test & ≥30% of the time post-cART VL detectable & ≥30% of the time post-cART CD4 <350 cells/mm^3^ | 28 | 232 | 14 | 22 | 67%  (53.2%-77.9%) | 91%  (88.5%-93.5%) |

^a^ TP- true positive , ^b^ TN- true negative ^c^ FN- false negative, ^d^ FP- false positive, ^e^ PPV- positive predictive value, ^f^ NPV- negative predictive value
